# Increased Risk of Long-Term Sickness Absence, Lower Rate of Return to Work, and Higher Risk of Unemployment and Disability Pensioning for Thyroid Patients: A Danish Register-Based Cohort Study

**DOI:** 10.1210/jc.2013-4468

**Published:** 2014-06-17

**Authors:** M. A. Nexo, T. Watt, J. Pedersen, S. J. Bonnema, L. Hegedüs, A. K. Rasmussen, U. Feldt-Rasmussen, J. B. Bjorner

**Affiliations:** The National Research Centre for the Working Environment (M.A.N., J.P., J.B.B.), DK-2100 Copenhagen, Denmark; Department of Public Health (M.A.N., J.B.B.), Section of Social Medicine, University of Copenhagen, Copenhagen DK-1014, Denmark; Department of Medical Endocrinology (T.W., A.K.R., U.F.-R), Copenhagen University Hospital (Rigshospitalet), Copenhagen DK-2100, Denmark; Department of Endocrinology and Metabolism (S.J.B., L.H.), Odense University Hospital, Odense DK-5000, Denmark; and QualityMetric (an Optum company) (J.B.B.), Lincoln, Rhode Island 02865

## Abstract

**Context:**

Little is known about how thyroid diseases affect work ability.

**Objective:**

The objective of this study was to evaluate the risk of work disability for patients with thyroid disease compared with the general population.

**Design, Setting, and Participants:**

In a longitudinal register study, outpatients (n = 862) with nontoxic goiter, hyperthyroidism, Graves' orbitopathy (GO), autoimmune hypothyroidism, or other thyroid diseases and their matched controls (n = 7043) were observed in the years 1994–2011 in Danish national registers of social benefits, health, and work characteristics. Cox regression analyses estimated adjusted hazard ratios (HRs) for the first year after diagnosis and subsequent years.

**Main Outcome Measures:**

Transitions between work, long-term sickness absence, unemployment, and disability pension were measured.

**Results:**

Patients differed significantly from the general population with regard to sickness absence, disability pension, return from sickness absence, and unemployment. In the first year after diagnosis, higher risks of sickness absence was seen for GO (HR 6.94) and other hyperthyroid patients (HR 2.08), who also had lower probability of returning from sickness absence (HR 0.62) and higher risk of disability pension (HR 4.15). Patients with autoimmune hypothyroidism showed a lower probability of returning from sickness absence (HR 0.62). In subsequent years, GO patients had significantly higher risk of sickness absence (HR 2.08), lower probability of return from sickness absence (HR 0.51), and unemployment (HR 0.52) and a higher risk of disability pension (HR 4.40). Hyperthyroid patients also had difficulties returning from sickness absence (HR 0.71).

**Conclusions:**

Thyroid patients' risk of work disability is most pronounced in the first year after diagnosis and attenuates in subsequent years. GO patients have the highest risk of work disability.

Recent years have witnessed increased interest in the short- and long-term impact of living with thyroid disease. Thyroid dysfunction has significant impact on somatic and psychiatric morbidity (1–4), and both short- and long-term health-related quality of life (HRQOL) is affected by a number of thyroid diseases (5–12). However, only a few studies have evaluated the impact on the work role function.

According to the health and disability model (13), the social consequences of diseases are understood as health-related barriers to the ability to participate in various social roles in society, including work. This model proposes that the impact of disease on work functioning, along with other individual and environmental factors, can lead to temporary or permanent exclusion from the labor market (eg, sickness absence, unemployment, or disability pensioning).

In studies of self-reported work role functioning, patients with newly diagnosed and untreated hyper- and hypothyroidism and nontoxic goiter have reported work role limitations on the SF-36 Health Survey (SF-36) role physical and role emotional scales (6). More than 30% of patients with hyperthyroidism have been reported to be completely or partially work disabled (14), although the extent of sickness absence seem most pronounced in the time after treatment initiation (15, 16). For Graves' orbitopathy, patients in remission reported a higher degree of work role limitations (SF-36) compared with the general population and patients with diabetes mellitus (17), and patients with active disease had a higher prevalence of work limitations and longer duration of sickness absence compared with the general population, healthy subjects, and patients with other thyroid or autoimmune diseases (18). However, these self-report studies are small and may suffer from selection bias due to patient nonresponse, information bias if patients under- or overassess their work ability, or confounding because of the lack of proper control of comorbidity.

In this study, we use registers to evaluate the risk of disease absence, unemployment, and disability pension in patients with thyroid diseases, using a cohort design with a follow-up of up to 17 years and a general population comparison group. We hypothesize that individuals with thyroid disease have a higher risk of long-term sickness absence, unemployment, and disability pension compared with individuals without thyroid disease, and we analyze the risk according to thyroid diagnostic subgroup. We distinguish between the risks in the first year after diagnosis and the risks in subsequent years, during which the disease is assumed to be under medical control or in remission.

## Materials and Methods

### Design

**Figure 1.**
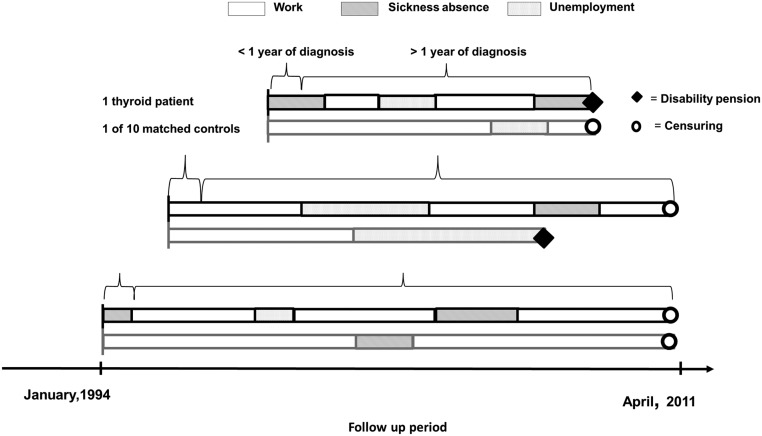
Examples of transitions between work-related states for patients with thyroid diseases and their matched controls.

Transitions between states of work, sickness absence, unemployment, and disability pension were observed in a population of patients with thyroid disease and controls from January 3, 1994, to April 1, 2011. We searched records back to 1977 to establish the earliest time of diagnosis. Patients with thyroid disease entered the analyses at the time of diagnosis or on January 3, 1994, if diagnosed before. For each patient, we identified 10 controls matched for age, gender, and region of habitation. The controls entered the analyses simultaneously with the thyroid patients. The risks of transitions between states in the first year after thyroid diagnosis and subsequent years were compared with that of the controls. The participants were censored at the end of the observation period (April 1, 2011), or before that point in time, if they died, turned 60 years old, or emigrated. Figure 1 illustrates the study design by showing examples of observed transitions between states of three patients and one of their matched controls in the follow-up period.

### Patient population

Patients with thyroid disease were recruited from two university hospital outpatient clinics in 2007 and 2008 (19). From the total sample of 1290 participants, the following patients were excluded: 250 below 18 or above 59 years of age at the start of the follow-up period (January 3, 1994), 43 who were pregnant when recruited for the study in 2007–2008, 84 on disability pension at the time of diagnosis or were receiving salary support because of reduced ability to work, 35 had died or emigrated, and 16 whose time of diagnosis could not be identified through available data sources. A total of 862 patients were included.

Information on diagnosis, date of diagnosis, and severity of disease was obtained through reviews of medical charts from the outpatient clinics. The participants were classified by initial diagnosis into five diagnostic categories (19):

1. Nontoxic goiter (normal thyroid hormone levels and normal TSH and palpable goiter verified by Tc-99m pertechnetate scan/ultrasonography).
2. Hyperthyroidism by either nodular toxic goiter [on Tc-99m pertechnetate scan/ultrasonography, suppressed TSH with (overt) or without (mild) elevated thyroid hormones, and absence of TSH receptor antibodies; Graves' hyperthyroidism: diffuse pattern on Tc-99m pertechnetate scan/ultrasonography, suppressed TSH with or without elevated thyroid hormone levels, and elevated TSH receptor antibodies, without signs of orbitopathy].
3. Graves' orbitopathy: Graves' disease and ophthalmopathy with a classification of no signs or symptoms; only signs, no symptoms; signs only; proptosis; eye muscle involvement; corneal involvement; sight visual acuity reduction performed by an ophthalmologist and Clinical Activity Scoring by an endocrinologist (20).
4. Autoimmune hypothyroidism: elevated serum TSH with (overt) or without (mild) decreased thyroid hormone levels and elevated levels of serum thyroperoxidase antibody and/or thyroglobulin antibody.
5. Other thyroid diseases (eg, postpartum thyroiditis, subacute thyroiditis, or other chronic or congenital thyroid diseases; see reference 19 for details).

The time of diagnosis was identified via three data sources: patient charts, the Danish National Patient Register (DIAG), and the Danish National Prescription Registry (PRESCRIBE) (21). The earliest date was applied, regardless of the data source.

DIAG includes registrations of all patients admitted to Danish hospitals since January 1977. The validity of the DIAG is high and studies found little misclassification of thyroid diseases (3, 22). PRESCRIBE provides information (including Anatomical Therapeutic Chemical classification codes, dates of dispensing, strength, and quantity) on all prescribed medications dispensed from Danish pharmacies since 1995. The codes H03AA01, H03AA02, H03BB01, H03BA02, and H03BB02 indicated thyroid treatment in this study.

### Control population

A population of 9000 randomly selected controls was drawn from the general population via the Danish Civil Registration system (23) and matched by age, gender, and regional area at Statistics Denmark. A total of 1957 individuals were excluded prior to the analyses; 598 received disability pension or salary support because of reduced work ability before entering the analyses (January 1, 1994), 279 had died or emigrated, 45 turned 60 years of age at study entry, the social reimbursements of 31 could not be identified via available data sources, 598 persons were identified in DIAG or PRESCRIBE with thyroid disease, and 406 were excluded because their matched thyroid patients were excluded. A total of 7043 controls remained.

### Outcome variables

Subjects were classified with sickness absence if receiving sickness absence benefits for a period of at least 3 weeks. Similarly, persons were classified as unemployed if receiving social benefits for unemployment. Persons receiving disability pension or salary support because of permanently reduced ability to work (Danish: Flex job) were categorized as receiving disability pension. Persons receiving no benefits were classified as self-supporting and consequently as working (24). These outcomes were identified via a national register of social public transfer payments: the Danish Register for Evaluation of Marginalization (DREAM).

DREAM provides weekly information on social transfer payments for all residents in Denmark (25). In Denmark, sickness absence that lasts for a minimum of 3 weeks is compensated by the Danish municipalities. Thus, DREAM contains complete registration of sickness absence for the follow-up period of the present study. DREAM includes all persons who have received social benefits or any other transfer income since July 1991. We used data from 1994, during which the register had achieved high quality. The registers included in this study are comprehensive and of high quality (21, 25). DREAM has been evaluated to be suitable for follow-up of social consequences of disease (25).

### Covariates

Eight covariates were included in all of the analyses, regardless of significance levels: 1) gender; 2) job type based on the International Standard of Classification of Occupations (26, 27) and condensed into three categories of increasing cognitive job demands (low, medium, and high); 3) household status (marital status and number of children); 4) number of working hours per week (above or below 29 hours); 5) immigrant status (Danish born or immigrant); 6) season (winter, spring, summer, autumn) and time period (1994–1996, 1997–1998, 1999–2000, 2001–2002, 2003–2004, 2005–2006, 2007, 2008–2011); 7) region of living (Sealand, Copenhagen, Southern Denmark); and 8) comorbidity.

Information on household status, working hours, and immigrant status were identified from national population and labor market registers at Statistics Denmark.

We included 32 chronic somatic and seven psychiatric diagnostic groups classified within the 22 domains of the *International Statistical Classification of Diseases and Related Health Problems*, 10th revision. Participants were identified as having a chronic disease via DIAG or PsychDIAG (DIAG's equivalent for psychiatric diseases) (21), by diagnostic codes of the *International Statistical Classification of Diseases and Related Health Problems*, 10th revision, or by prescribed medication (eg, insulin) via PRESCRIBE. We did not control for thyroid disease or eye diseases because an eye disease could be a consequence of having a thyroid disease.

### Statistical analyses

Five transitions between the work related states were analyzed in a multistate model (24): 1) from work to sickness absence; 2) from sickness absence to work; 3) from work to unemployment; 4) from unemployment back to work; and 5) from work, sickness absence, or unemployment to disability pension.

The transition from sickness absence to unemployment was not analyzed because there were too few instances. Each transition was analyzed separately using the Cox proportional hazard model.

The overall impact of thyroid disease was evaluated through a likelihood ratio (LR) test. We estimated hazard ratios (HRs) separately for each disease subgroup and evaluated the impact of thyroid disease by excluding patients with Graves' orbitopathy. Separate tests were performed and HRs estimated of the transitions within the first and subsequent years of thyroid disease. The underlying time axis in the analyses was age. Work, sickness absence, and unemployment were treated as transient states, meaning that the participants could leave and reenter these states (24). The transition to disability pension (from work, sickness absence, or unemployment) was regarded as an absorbing event, in which participants were not at risk for further transitions. Participants on, for example, maternity leave or studying were temporarily excluded from the model until they returned to work, sickness absence, or unemployment. All variables except thyroid disease, gender, and region were treated as time-dependent variables. Assumptions of proportional hazards were visually evaluated by cumulative hazard curves. All statistical analyses were performed in SAS version 9.2.

We tested whether analyzing nodular toxic goiter and Graves' hyperthyroidism as separate clinical groups would improve the model. Because the LR tests did not find significant differences between work-related outcomes for these groups, we present only the results for the combined group: hyperthyroidism. The outcomes for patients with Graves' orbitopathy differed significantly from the other hyperthyroid patients, so these results are presented separately. Exclusion of thyroid patients from the control sample resulted in nearly identical results.

A written consent from patients included in this study was obtained by medical staff (19). All the databases were hosted and linked at Statistic Denmark. The study was registered and approved by the Danish Protection Agency, identification number: 2010-41-5542.

## Results

### Sample characteristics, events and length of observation

At study entry and exit, more thyroid patients suffered from nonthyroidal comorbidities, compared with the general population sample (Table 1). Also, more thyroid patients were single and living in households without children. Table 2 presents the observation time in person years and the number of events for each of the five transitions. Few events were observed for transitions toward disability pension in the first year after diagnosis of thyroid disease, and patients with Graves' orbitopathy and other thyroid diseases had no events for this transition (Table 2).

**Table 1.** Characteristics of Patients and Controls When Entering the Study

	Thyroid Population		General Population	
	n = 862	%	n = 7043	%
Age, y				
20–29	155	18	1349	19
30–39	255	30	2184	31
40–49	234	27	1935	27
50–59	218	25	1575	23
Gender				
Female	765	89	6210	88
Male	97	11	833	12
Immigrant status				
Immigrant	91	11	605	9
Danish	771	89	6438	91
Household status				
Single, no children	224	26	1386	20
Single with children	71	8	541	8
Cohabitants, no children	266	31	2208	31
Cohabitants with children	293	34	2842	40
Missing	8	1	66	1
Cognitive job demands				
High	169	20	1174	17
Medium	315	36	2537	36
Low	377	44	3307	47
Missing	1	<1	25	<1
Weekly working hours				
Above 29 h	423	49	3500	50
Below 29 h	348	40	2749	39
Missing	91	11	794	11
Regional area				
Copenhagen	479	55	3967	56
Southern Denmark	344	40	2745	39
Sealand	39	5	331	5
Thyroid diagnosis				
Nontoxic goiter	270	31		
Hyperthyroidism	262	32		
Graves' orbitopathy	76	9		
Hashimoto's thyroiditis	176	20		
Other thyroid diseases	78	9		
Year of Diagnosis				
Before 1994	102	11		
1994–2000	170	20		
2001–2006	377	44		
2007–2009	213	25		
Comorbidities^a^				
None	446	52	4182	59
When entering the study				
1	219	25	1634	23
2 or above	197	23	1227	18
Comorbiditides^a^				
None	234	27	2659	38
When exiting the study				
1	244	28	1998	28
2 or above	384	45	2386	34

a Number of nonthyroidal diseases per person.

**Table 2.** Events and Person Years for the Thyroid and Control Populations in Each of the Five Work Transitions

	Sickness Absence		Return to Work From (SA)		Unemployment		Return to Work From U		Disability Pension	
	Events	PY	Events	PY	Events	PY	Events	PY	Events	PY
Nontoxic goiter										
<1 y	27	161	31	10	56	161	55	28	6	198
>1 y	142	1320	138	63	488	1320	500	212	14	1595
Hyperthyroidism										
<1 y	33	138	40	20	32	138	38	28	7	186
>1 y	142	1255	130	76	376	1255	381	210	15	1540
Graves' orbitopathy										
<1 y	26	38	23	10	4	38	4	7	0	55
>1 y	68	320	62	45	63	320	63	72	20	437
Autoimmune hypothyroidism										
<1 y	17	107	22	13	29	107	30	17	2	138
>1 y	100	638	91	52	171	638	165	96	12	786
Other thyroid disease										
<1 y	9	40	11	4	19	40	17	14	0	57
>1 y	51	278	48	21	82	278	83	96	5	395
Entire thyroid population										
<1 y	112	485	127	57	140	485	144	94	15	634
>1 y	503	3810	469	256	1180	3810	1192	685	76	4753
Control population										
<1 y	525	4506	458	184	1113	4506	1187	650	39	5340
>1 y	3505	35089	3194	1399	7664	35089	7826	4545	369	41033

Abbreviation: PY, person years; SA, sickness absence; U, unemployment.

### Transitions from work to long-term sickness absence

Within the first year of diagnosis, thyroid patients differed significantly from controls (*P* < .0001) and also in tests excluding Graves orbitopathy (*P* < .02, Table 3). Patients with hyperthyroidism (HR 1.96) and patients with Graves' orbitopathy (HR 6.94) had significantly higher risk of long-term sickness absence than the control population.

**Table 3.** Probability of the Five Work Transitions for Patients With Different Thyroid Diagnoses Compared With the Control Population

LR Tests (5 Degrees of Freedom)	Sickness Absence		Return to Work From SA		Unemployment		Return to Work From U		Disability Pension	
	Chisquare	*P* Value	Chisquare	*P* Value	Chisquare	*P* Value	Chisquare	*P* Value	Chisquare	*P* Value
Thyroid patients vs controls										
<1 y	**67.0**	<**.0001**	**17.1**	**.0043**	4.3	.5092	4.7	.4589	**525.9**	<**.0001**
>1 y	**24.6**	**.0002**	**32.6**	<**.0001**	10.1	.0719	**17.2**	**.0042**	**35.5**	<**.0001**

HR	HR	(95% CI)	HR	(95% CI)	HR	(95% CI)	HR	(95% CI)	HR	(95% CI)
Nontoxic goiter										
<1 y	1.23	(0.80–1.89)	1.37	(0.87–2.15)	1.34	(0.79–2.27)	1.02	(0.58–1.81)	3.67	(1.43–9.41)
>1 y	0.93	(0.77–1.13)	1.10	(0.84–1.43)	**1.48**	**(1.11–1.99)**	1.31	(0.98–1.73)	0.71	(0.43–1.17)
Hyperthyroidism										
<1 y	**1.96**	**(1.31–2.94)**	**0.62**	**(0.43–0.89)**	1.01	(0.61–1.66)	0.78	(0.52–1.19)	**4.15**	**(1.76–9.77)**
>1 y	1.14	(0.91–1.42)	**0.71**	**(0.53–0.95)**	1.46	(0.91–2.33)	1.12	(0.68–1.83)	1.12	(0.64–1.96)
Graves' orbitopathy										
<1 y	**6.94**	**(4.19–11.50)**	0.63	(0.33–1.19)	0.49	(0.14–1.78)	0.40	(0.14–1.16)	^a^	^a^
>1 y	**2.08**	**(1.48–2.93)**	**0.51**	**(0.40–0.66)**	0.91	(0.49–1.69)	**0.52**	**(0.37–0.75)**	**4.40**	**(2.61–7.42)**
Autoimmune hypothyroidism										
<1 y	1.36	(0.82–2.28)	**0.56**	**(0.36–0.86)**	1.10	(0.57–2.12)	0.95	(0.52–1.74)	2.46	(0.63–9.65)
>1 y	1.26	(0.91–1.73)	0.79	(0.56–1.11)	1.17	(0.79–1.74)	0.89	(0.67–1.18)	1.49	(0.81–2.74)
Other thyroid disease										
<1 y	1.61	(0.77–3.35)	1.52	(0.57–4.10)	1.72	(0.80–3.70)	0.81	(0.44–1.47)	^a^	^a^
>1 y	1.69	(0.98–2.88)	1.10	(0.66–1.85)	1.10	(0.61–1.99)	0.87	(0.51–1.48)	0.48	(0.12–1.99)

LR Tests (4 Degrees of Freedom)	Chisquare	*P* Value	Chisquare	*P* Value	Chisquare	*P* Value	Chisquare	*P* Value	Chisquare	*P* Value
Thyroid patients (excluding GO) vs controls										
<**1 y**	**13.0**	**.0115**	**17.4**	**.0016**	3.08	.5451	1.9	.7598	**328.7**	<**.0001**
>1 y	7.2	.1236	7.9	.0967	**10.0**	**.0408**	4.6	.3269	4.7	.3152

Abbreviation: CI, confidence interval; GO, Graves' orbitopathy; SA, sickness absence; U, unemployment. Statistically significant estimates shown in bold.

a Too few instances to allow for estimation.

In subsequent years, thyroid patients differed significantly from the control population (*P* < .0002 overall) primarily due to a higher risk of long-term sickness absence for patients with Graves' orbitopathy (HR 2.08).

### Transition from long-term sickness absence to work

Within the first year of diagnosis, thyroid patients differed significantly from controls (*P* < .005 overall and *P* < .002 excluding Graves' orbitopathy). Significantly lower HRs of return to work was found in patients with hyperthyroidism (HR 0.62) and autoimmune hypothyroidism (HR 0.56). A low HR (<1) indicates a low probability of returning to work and thus a relatively long sick leave.

In subsequent years, thyroid patients differed significantly from controls (*P* < 001 overall), primarily due to lower probability of return to work for patients with Graves' orbitopathy (HR 0.51) and other forms of hyperthyroidism (HR 0.71).

### Transition from work to unemployment

No significant difference between thyroid patients and controls was found in the first year after diagnosis. In subsequent years, a significant difference was found only for comparisons that excluded patients with Graves' orbitopathy, primarily driven by a higher risk of unemployment for patients with nontoxic goiter (HR 1.48).

### Transition from unemployment to work

In the first year after diagnosis, no significant difference was found between thyroid patients and controls.

In subsequent years, thyroid patients differed from the general population (*P* < .005 overall), primarily due to a lower probability of return to work for patients with Graves' orbitopathy (HR 0.52).

### Transition from work, sickness absence, and unemployment to disability pension

Within the first year of diagnosis, thyroid patients differed significantly from controls (*P* < .0001 overall and *P* < .0001 excluding patients with Graves' orbitopathy). Patients with hyperthyroidism had significantly higher risk of disability pension (HR 4.15). During subsequent years, thyroid patients and controls differed significantly (*P* < .0001 overall), primarily due to a higher risk of disability pension among patients with Graves' orbitopathy (HR 4.40).

## Discussion

Patients with Graves' orbitopathy generally had the most negative outcomes. Graves' orbitopathy patients had a 7 times increased risk of sickness absence within the first year of diagnosis, and twice the risk during subsequent years, along with half the probability of returning to work after sickness absence or unemployment. Due to small numbers, the risk of disability pension among patients with Graves' orbitopathy could not be estimated in the first year after diagnosis, but in the following years, the risk was more than 4 times as high as among controls. These results are in line with reports from previous studies that Graves' orbitopathy impacts quality of life (9, 28), including work role functioning (17) and sickness absence (18). The present study suggests that in addition to the higher risk of sickness absence, socioeconomic costs of Graves' orbitopathy also entail difficulties getting back to work from sickness absence or unemployment and increased risk of disability pension.

The group of patients with hyperthyroidism had twice the risk of sickness absence within the first year of diagnosis and a lower probability of return to work in both the first and in subsequent years. This is in line with previous studies, in which 30% or more of patients with hyperthyroidism reported temporary or permanent work disabilities (14) and reporting that sickness absence was most pronounced after treatment initiation (15, 16). To increase statistical power, we grouped patients with toxic nodular goiter and patients with Graves' hyperthyroidism despite different disease mechanisms. Patients with Graves' disease typically have more severe hyperthyroidism (29) than patients with toxic nodular goiter and the causes of morbidity and mortality consequently differ (2, 30) as may the impact on HRQOL (29). For example, one HRQOL study showed lower scores on vitality, work role functioning, and mental health scales for patients with Graves' hyperthyroidism 14–21 years after treatment initiations (5). Despite these different disease mechanisms, initial analyses and formal testing of this study showed that the two groups had similar work-related outcomes.

We did not observe strong indications of work disability in patients with autoimmune hypothyroidism, except for a significantly diminished probability of return to work after sickness absence in the first year after diagnosis. Previous literature has shown an increased psychiatric comorbidity in hypothyroid patients (3, 4), and studies using self-reported outcomes found an impact on several HRQOL dimensions including persistent fatigue, sleep disturbances, or lack of concentration (8, 31–33) and work role functioning (6). It is possible that patients with autoimmune hypothyroidism may generally be able to compensate for their symptoms at work and avoid sick leave and early retirement despite their perceived problems.

The results regarding the risk of unemployment were less clear than the results regarding sickness absence and disability pension. A possible explanation is that the risk of unemployment is influenced by other factors, some of which, eg, workplace closure, are beyond the influence of employee-related factors. An unexpected finding was the increased risk of unemployment in patients with nontoxic goiter. However, because the overall test did not show a significant difference between thyroid patients and the general population, the increased risk of unemployment in patients with nontoxic goiter may be a spurious result. Although lowered HRQOL has been reported among patients with newly diagnosed nontoxic goiter (6), the extent of long-term disability of this patient group is unknown and future research is needed.

A main strength of the current study is the prospective design, enabling investigation of the risk of different work-related outcomes over time. The existence of high-quality registers of social benefits linked through unique personal identification numbers enables a high degree of follow-up and a low risk of misclassification bias. In Denmark, the right to receive sickness absence benefits is regulated by law and not dependent on membership of an insurance scheme and Danish citizens have easy access to unemployment benefits (25, 34). Because thyroid diseases vary greatly in severity, duration, and manifestations, we decided to analyze data by clinical phenotype. This strategy provided less statistical power but gave a more detailed, valid, and clinically relevant description. A further strength is the distinction between risks during the first year after diagnosis and risks in subsequent years. In terms of providing prognostic information to patients, it is of value to be able to differentiate between impact on work life during the first year, when treatment is initiated, and on work life in subsequent years. Finally, the multistate analysis allowed simultaneous analyses of change between several states. This approach is well suited for analysis of work-related outcomes such as sickness absence, unemployment, and disability pension because they represent competing outcomes.

Among the limitations of the current study is the small sample size for some of the thyroid diseases, resulting in wide confidence intervals. Also, we could not estimate the risk for some rare transitions, eg, from sickness absence to unemployment. A larger sample size could be achieved by identifying the patient population by registers, but this would give a far less valid identification and classification of thyroid disease. In addition, in few instances, unemployment benefits cannot be received (eg, if a spouse has a high income), and in these cases a person would be registered as self-supporting in the DREAM register rather than unemployed.

The clinical data were recorded only at study entry, and we had no information on clinical changes in the thyroid disease over time, eg, development of orbitopathy in patients with Graves' hyperthyroidism. However, we did update information on acquired comorbidities throughout the entire follow-up period and adjusted for comorbidities in all of the analyses. The patients were recruited from hospitals and may represent patients with more severe diseases compared with patients treated in general practice. Finally, patients were censored when turning 60 years old because many Danes become eligible for early retirement benefit at this age. Thus, our results pertain only to patients younger than 60 years.

Risks of sickness absence, unemployment, and disability pension may differ between countries. The Danish labor market is characterized as a flexicurity system. The welfare state secures easy access to social benefits, but Denmark has relatively little formal employment protection, allowing, for instance, an employer to lay off an employee with long-term sickness absence. The economic recession of 2007–2008 may provide particular employment challenges for persons with chronic disabilities (35). Although our analyses controlled for time period and thus adjusted for the general impact of the recession, the analyses may have missed a specific impact on patients with thyroid disease.

In line with previous research, this study documents that thyroid diseases impact the ability to work, but the extent of work disability depends on the type and length of thyroid disease. Graves' orbitopathy patients had the highest risks of temporary and permanent work disability. The risk of work disability also extended beyond the first year for other patients with hyperthyroidism but to a smaller extent than Graves' orbitopathy patients. The work disability observed in patients with autoimmune hypothyroidism was limited to the first year of diagnosis. These results stress the importance of clinicians inquiring into the work function of their patients as part of their clinical assessment. However, it is also important to note that the potential problems seem to be most pronounced within the first year of diagnosis and diminish with time, presumably because of treatment effects and/or effective coping by patients As an example, the invalidating cognitive dysfunction seen in Graves' hyperthyroidism seemed to attenuate with treatment and length of follow-up (36). Regardless, the evidence of improvement with time is an important message for patients and their health care providers.
